# Non-nucleosidic inhibition of Herpes simplex virus DNA polymerase: mechanistic insights into the anti-herpetic mode of action of herbal drug withaferin A

**DOI:** 10.1186/1471-2105-12-S13-S22

**Published:** 2011-11-30

**Authors:** Abhinav Grover, Vibhuti Agrawal, Ashutosh Shandilya, Virendra S Bisaria, Durai Sundar

**Affiliations:** 1 Department of Biochemical Engineering and Biotechnology, Indian Institute of Technology (IIT) Delhi, Hauz Khas, New Delhi 110016, India; 2 Supercomputing Facility for Bioinformatics and Computational Biology, Indian Institute of Technology (IIT) Delhi, Hauz Khas, New Delhi 110016, India

## Abstract

**Background:**

Herpes Simplex Virus 1 and 2 causes several infections in humans including cold sores and encephalitis. Previous antiviral studies on herpes viruses have focussed on developing nucleoside analogues that can inhibit viral polymerase and terminate the replicating viral DNA. However, these drugs bear an intrinsic non-specificity as they can also inhibit cellular polymerase apart from the viral one. The present study is an attempt to elucidate the action mechanism of naturally occurring withaferin A in inhibiting viral DNA polymerase, thus providing an evidence for its development as a novel anti-herpetic drug.

**Results:**

Withaferin A was found to bind very similarly to that of the previously reported 4-oxo-DHQ inhibitor. Withaferin A was observed binding to the residues Gln 617, Gln 618, Asn 815 and Tyr 818, all of which are crucial to the proper functioning of the polymerase. A comparison of the conformation obtained from docking and the molecular dynamics simulations shows that substantial changes in the binding conformations have occurred. These results indicate that the initial receptor-ligand interaction observed after docking can be limited due to the receptor rigid docking algorithm and that the conformations and interactions observed after simulation runs are more energetically favoured.

**Conclusions:**

We have performed docking and molecular dynamics simulation studies to elucidate the binding mechanism of prospective herbal drug withaferin A onto the structure of DNA polymerase of Herpes simplex virus. Our docking simulations results give high binding affinity of the ligand to the receptor. Long *de novo* MD simulations for 10 ns performed allowed us to evaluate the dynamic behaviour of the system studied and corroborate the docking results, as well as identify key residues in the enzyme-inhibitor interactions. The present MD simulations support the hypothesis that withaferin A is a potential ligand to target/inhibit DNA polymerase of the Herpes simplex virus. Results of these studies will also guide the design of selective inhibitors of DNA POL with high specificity and potent activity in order to strengthen the therapeutic arsenal available today against the dangerous biological warfare agent represented by Herpes Simplex Virus.

## Background

Herpes Simplex Virus type 1 and type 2 (HSV-1 and HSV-2) are two members of *Herpesviridae* family, which infect almost 85% of the world population [1]. They are the causative agents of a gamut of diseases ranging from mild ones like cold sores in mouth, eye cornea and genitals to more severe life threatening ones like the fatal herpes encephalitis [2]. People with suppressed immune system like those suffering from AIDS are more prone to get infections from HSV than others [3].

### Drugs for HSV

There is no permanent cure for these infections till date. Present day treatment involves the use of antiviral drugs to reduce the physical severity of outbreak-associated lesions and viral shedding, though this helps decreasing the chances of transmission to others only by maximum 50% [4]. There are two types of drugs that are clinically useful against HSV infections. The first category consists of nucleoside analogs like acyclovir and its prodrug valacyclovir, ganciclovir, penciclovir and its prodrug famciclovir, sorivudine and brivudine. These require phosphorylation by viral thymidine kinase to form triphosphates that are active inhibitors of viral DNA polymerase. The second category consists of direct viral DNA polymerase inhibitors like vidarbine, foscarnet and cidofovir [5]. Thus, both types of drugs target in dysfunctioning the replication centre i.e. DNA polymerase of the viral genome [6].

### Development of alternative treatments for HSV

However, in past few years, a number of acyclovir drug resistant viral strains have been isolated especially from immuno-compromised patients [7-9]. In this era, where the number of immuno-suppressed patients like those suffering from HIV is continuously increasing, there is an immediate need to find new drugs to treat HSV infections which have a higher efficacy or have an alternative mode of action [10]. Resistance to acyclovir is mainly due to mutations in the viral thymidine kinase (TK) gene which impair the initial drug phosphorylation [11]. These drug resistant strains have been of significant clinical attention [12,13], indicating the need for alternative anti- HSV drugs. Previous antiviral studies on herpes viruses have focussed on developing nucleoside analogues that will inhibit viral polymerase and terminate the replicating viral DNA. A number of new anti-viral drugs against HSV DNA polymerase are currently under research and development; these focus other domains of the polymerase than those targeted by the commercially available drugs [14]. One such novel class of compounds is that of 4-oxo-DHQs belonging to the non-nucleoside anti-herpetic drugs family [15].

### Traditional medicines

Medicinal plants products have been used over centuries as traditional remedies for different kinds of diseases including viral diseases. Recently, there have been studies which report anti-viral activities of extracts from plants like *Swertia chirata*, *Aloe forex* and *Withania somnifera* against HSV [16,17]. These plant extracts inhibit the formation of HSV-1 plaque above a certain minimum concentration and their activities can be compared to the commercial drugs like acyclovir.

*Withania somnifera* or Winter Cherry or Indian ginseng is a proud herb of Ayurveda, classified as Rasayan (the most esteemed of Ayurveda herbs) [18]. It is held in high repute in traditional Indian medicine mainly because of its constituents called withanolides [19]. They are built on an ergostane framework, which is oxidized at C-22 and C-26 to form a six-member lactone ring. Withaferin A (WA), the first withanolide to be isolated and the major withanolide present in Indian variety of plants has been widely researched for its pharamacological activities including anti-inflammatory, anti-cancer, anti-stress and immunomodulatory, adaptogenic, central nervous system, endocrine and cardiovascular activities [20-25]. Leaves of *Withania somnifera* have been reported to have the highest content of WA (around 0.001 to 0.5% dry weight of leaves) [26].

In this study, we report a possible mode of action of withaferin A against HSV by inhibition of its DNA polymerase. Molecular docking studies have been used to identify the binding modes. Dynamic structural patterns were studied using Molecular dynamics simulations.

## Methods

### Ligand and receptors

**Figure 1:**
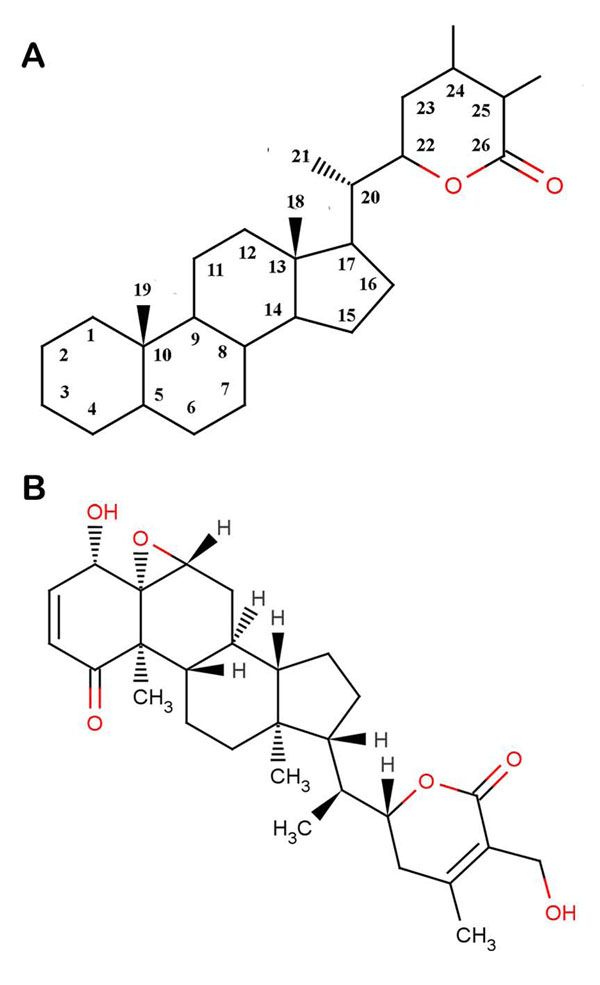
**Structures of withanolides.** (A) Withaferin A falls under the family of naturally occurring C28- steroidal lactones known as withanolides. (B) Structure of withaferin A.

The crystal structure of HSV DNA polymerase [PDB: 2GV9] was obtained from the Protein Data Bank (PDB) [27]. The structure of this enzyme has been described elsewhere [15]. This structure was then subjected to certain modifications which would make it suitable for docking as described elsewhere [28]. The structure of the ligand molecule withaferin A [PubChem:265237] retrieved from NCBI-PubChem Compound database [29] is shown in Figure 1. Amber 11 [30], was used to minimise the energy of the ligand and the receptor using Steepest Descent and Conjugate Gradient Methods.

### Semi-flexible docking

Semi-flexible molecular docking of the HSV DNA polymerase along with ligand WA was implemented using AutoDock 4.0 [31]. The general procedure for performing docking is described elsewhere [28]. The outputs from AutoDock were rendered with PyMOL [32] and Accelerys ViewerLite 5.0. Confirmation of the results were achieved using ParDOCK [33].

### MD simulations in water

The energy minimization and MD simulations of HSV POL and its complex with WA were carried out using AMBER package as fully described elsewhere by the authors [28].

## Results and discussion

### Docking of withaferin A into HSV POL

**Figure 2:**
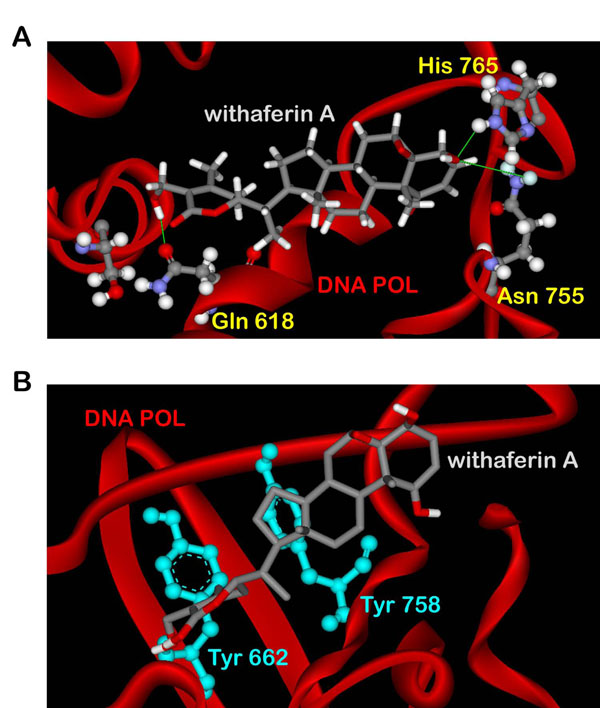
**Interactions of docked withaferin A with HSV POL before MD.** (A) H-Bond interactions of the docked ligand with the polymerase residues. (B) Docked withaferin A forming van der waals interactions with the hydrophobic residues of HSV POL.

We performed docking of WA into X-Ray crystal structure of HSV POL. Using binding pocket analysis, a cavity around the active site/subsites of HSV POL was predicted where WA was found binding to the critical active site residues of HSV POL with a binding energy of -8.46 Kcal/mol. The ligand formed several H-bond interactions with the crucial residues of the polymerase (Figure 2A). Ring amino hydrogen of His 765 formed bond with terminal hydroxyl group of WA. The same hydroxyl of WA bonded up with amino group of Asn 755 also. The other terminal hydroxyl of WA also interacted with side chain carbonyl of Gln 618. Gln 618 is one of the active site residues of HSV POL, being a part of the hydrophobic pocket. Apart from H-bonds, WA was also found forming hydrophobic interactions. As shown in Figure 2B, WA formed van der waals interactions with the highly conserved residues Tyr 662 and Tyr 758, which lie in close proximity to the catalytic site. The properties of the docked ligand are shown in Table 1.

**Table 1:** Properties of the docked conformation

Ligand	withaferin A
Docked using	AutoDock
Binding energy	-8.46 Kcal/mol
Ligand efficiency	-0.25
Inhibition constant	624.75 nM
Intermolecular energy	-9.03 Kcal/mol
Total internal energy	-0.8 Kcal/mol

### MD simulations in water

**Figure 3:**
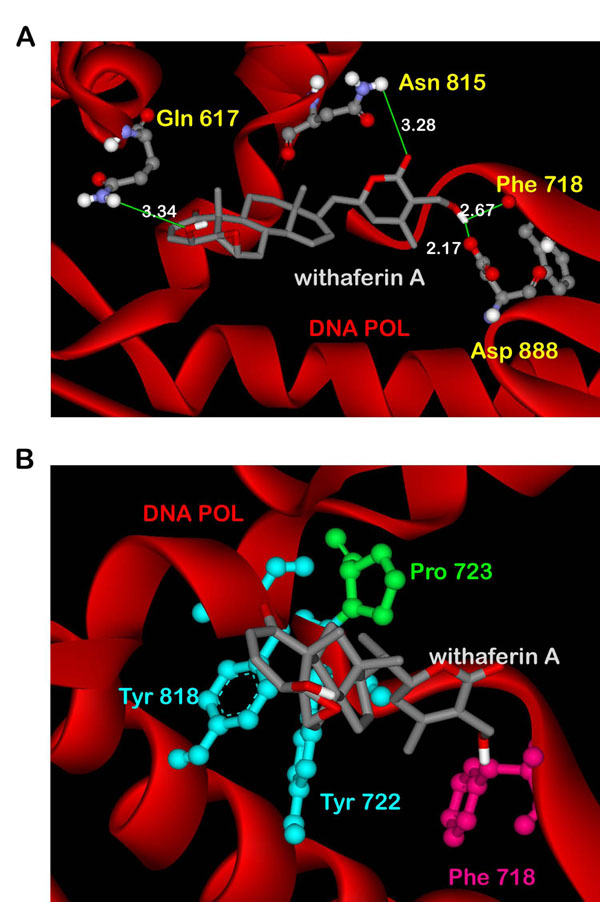
**Interactions of docked withaferin A with HSV POL post-MD.** (A) H-Bond interactions of the docked ligand with the polymerase residues. (B) Docked withaferin A forming van der waals interactions with the hydrophobic residues of HSV POL.

Since molecular docking provides only a static view of the protein-ligand interactions, we performed molecular dynamics simulations on HSV POL in complex with WA to study the interactions in motion. The overall goal of these simulation steps was to account for protein flexibility and movement that however could not be achieved in the docking simulations alone. Figure 3A shows the H-bond interactions of the docked WA with HSV POL in a 10 ns simulated snapshot. WA was found forming H-bond interactions with Gln 617, Phe 718, Asn 815 and Asp 888 of HSV POL. Strong hydrophobic interactions by the residues Phe 718, Pro 723, Tyr 722 and Tyr 818 of HSV POL were also observed in this simulated structure (Figure 3B).

**Figure 4:**
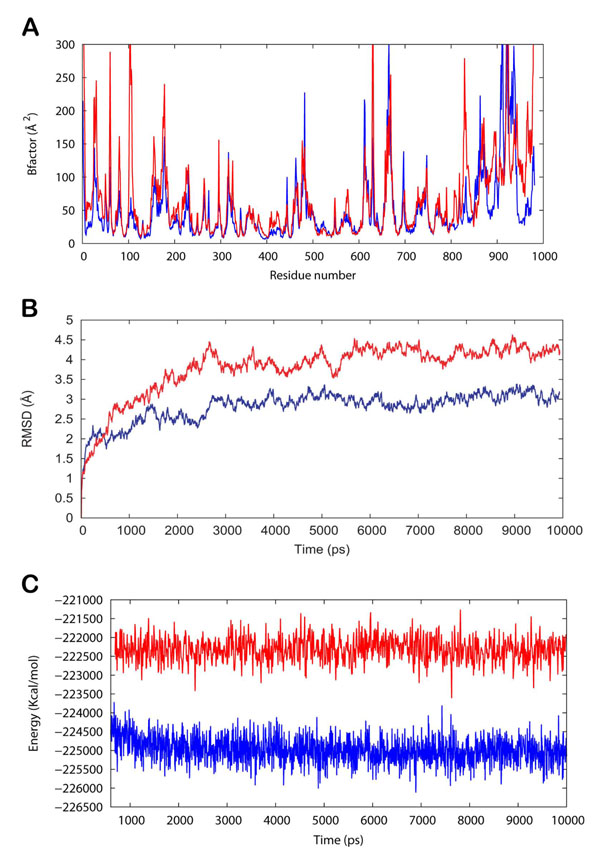
(A) Plot of B-factor values of HSV POL (red) and WA/HSV POL (blue). (B) Plot of root mean square deviation (RMSD) of Cα of HSV POL (protein) and WA/HSV POL (complex). (C) Plot of total energy of HSV POL (protein) and WA/HSV POL (complex).

To probe the dynamic flexibility changes in the protein, due to the inhibitor binding in its critical regions, B-factors (B_n_) for the Cα atoms were calculated using the following relation:

B_n_ = 8/3 π r_n_^2^

where r_n_ is the root-mean-square atomic fluctuation of the Cα atom of residue n. As shown in Figure 4A, the calculated B_n_ values for the undocked protein show a major peak for the residues 615-625 close to the active site that possibly determines its function through large-amplitude motion. However, on docking with WA, a decrease in B_n_ values of the protein was observed that shows its conformational rigidity. Further, this points out that the large-amplitude motion of the flexible residues 615-625, which encompasses two consecutive catalytically-active glutamines 617 and 618, is retarded by the binding of WA to active site of HSV POL. This suggests the inhibitory activity of WA against HSV POL, as in our docked structures WA established hydrogen bonds with these residues. In another instance, for the region 800-850 in which most of the catalytic site residues are located, B_n_ values for WA bound polymerase were seen attenuated in comparison to those of the enzyme alone. The RMSDs for the trajectory of WA bound HSV POL reveals the stability of the complex (Figure 4B). The RMSD of the complex has achieved a stationary phase during the later stage of the simulation and is always less than 3.0 Å for the entire simulation length. The thermodynamic stability of the complex was evident as the energy of the complex (blue) was found always lower than that of the protein (red) throughout the simulation (Figure 4C).

### Analysis of pre- and post-MD simulated structures

**Figure 5:**
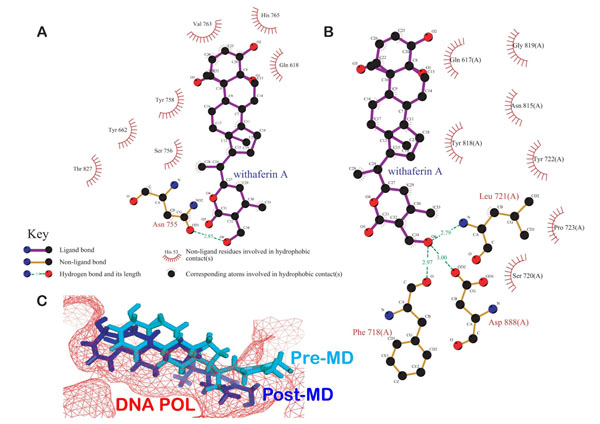
**Comparative analysis of pre- and post-MD simulated structures. (A) Ligplot of pre-MD structure (B) Ligplot of post-MD structure (C) Structural alignment of the ligand WA present in both structures**. WA slides down to acquire a more structurally stable configuration by anchoring its tail inside the gorge of HSV POL.

From the interactions shown in Figure 5A and 5B as Ligplots [34], a significant difference in the number and nature of interactions was observed. The binding energy based on the physico-chemical properties of the active site as well as that of the ligand were calculated as -11.45 and -8.84 Kcal/mol for the post- and pre-MD simulated structures respectively. The comparative parametric values of the two structures calculated using RASPD server [33] are shown n Table 2. Close visual inspection of the MD results permitted us to observe that post-MD, WA was able to establish a better structurally stable conformation. As evident from Figure 5C, in the pre-MD structure WA was not able to anchor appropriately its tail inside the cavity of HSV POL. On the other hand, the behaviour of WA was much more stable in the post-MD simulated structure where its tail is well anchored inside the deep narrow gorge of the polymerase. Moreover, the ligand substantially moved towards the residues of HSV POL to form additional H-bonds. The residues Phe 718 and Asp 888 with which WA established H-bonds in the post-simulated structure participate in metal-ion coordination essential for the catalysis of polymerase. Another WA H-bonding residue Asn 815 of HSV POL, is a highly conserved residue which besets on the prolonged second α helix of the finger domain. The continuous nature of this structure is responsible for bringing the other highly conserved residues Tyr 818 and Gly 819 into close proximity of the catalytic site for facilitating proper functioning of the polymerase [15]. In the post-MD simulated structure, the intensity of hydrophobic interactions was also found enhanced as compared to those present before MD. The residues Phe 718, Tyr 722, Pro 723 and Tyr 818 provided significant hydrophobic contacts for stabilization of WA inside the cavity. The ribose of the incoming nucleotide has been earlier report to interact with the conserved Tyr 722 residue and induces a steric access effect against inclusion of the nucleotides [15].

**Table 2:** Comparison of different parameters of docking of withaferin A onto DNA POL in pre- and post- MD simulated structures

Status	Pre-MD	Post-MD
Binding energy	-8.84 Kcal/mol	-11.45 Kcal/mol
H-Bond donors	20	23
H-bond acceptors	16	17
log P	48.47	24.84
Molar refractivity	535.3	295.09
ln (Vol)	8.15	8.08

### Non-nucleosidic inhibition of HSV POL by WA

It has been earlier reported that 4-oxo-dihydroquinolines (4-oxo-DHQs) have shown broad anti-HSV activity [35,36]. This class of compounds inhibit most human herpes viruses, which is associated with DNA polymerase inhibition. From the binding assays of PNU-183792 , a radiolabelled DHQ on HSV POL, it was observed that this inhibitor binds only to HSV POL in complex with the DNA duplex; while no binding was observed either with HSV POL or with DNA duplex alone [15]. However, in our case we observed that WA is able to bind to the segregated HSV POL itself. From visual inspection of the structures of WA and the 4-oxo-DHQ inhibitor, it was observed that both these ligands contain 2 ketonic groups, an oxygen containing heterocyclic ring and a tail containing an electronegative atom. In the present study, the binding mode of WA was found similar to that of the radiolabelled 4-oxo-DHQ in which the residues Gln 617, Gln 618, Asn 815 and Tyr 818 play critical role in the stabilization of the ligand. It can be deduced from our studies that WA can be a potent non-nucleosidic inhibitor of HSV POL whose binding would result in a conformational change in the polymerase that distorts the positioning of the residues that bind DNA, inhibiting polymerization. 4-oxo-DHQs have shown high specificity index in inhibiting DNA polymerases belonging to the *herpesviridae* family because unrelated DNA and RNA viruses were not susceptible to their inhibitory effect, and they also proved to have broad spectrum antiviral effects [35,36]. The same can be expected for WA owing to its analogous mode of action. The inhibition constant of WA found in our study (0.6 µm) was also quite comparable to the currently established nucleosidic drugs (0.1-0.6 µm) [37].

The emergence of HSV resistance to antiviral drugs is also a major concern. Three basic mechanisms have been identified: altered thymidine kinase substrate specificity, absent or partial production of viral thymidine kinase and altered viral DNA polymerase [10]. The most common mechanism found in clinical isolates is deficient TK activity. For the foremost approved drug- Acyclovir, resistant isolates of HSV have been observed in immuno-compromised individuals, especially AIDS patients [37]. Since WA is observed exerting its inhibitory effect via interaction with a viral DNA polymerase site that is less important for the binding of deoxynucleoside triphosphates, it holds potential to exert its influence even on these resistant isolates. The non-nucleosidic mode of action of WA holds promise for prevention of infection, as it can selectively target only the viral enzymes. Moreover being a naturally occurring herbal drug candidate, WA will also be able to address the issue of safety and bioavailability.

Based on the results from WA-HSV POL complex, it appears that interactions with the residues Gln 617, Gln 618, Tyr 722, Asn 815 and Asp 888 of HSV POL are important for inhibitory activity of WA. A comparison between the conformation obtained from docking and that from molecular dynamics simulations show that substantial changes in binding conformations have occurred. These results indicate that the initial receptor-ligand interaction observed after docking can be limited due to the receptor rigid docking algorithm and that the conformations and interactions observed after simulation runs are more energetically favoured and should be better representations of derivative poses in receptor.

## Conclusions

We have performed docking and molecular dynamics simulation studies to elucidate the binding mechanism of prospective herbal drug withaferin A onto the structure of DNA polymerase of Herpes simplex virus. Our docking simulations results give high binding affinity of the ligand to the receptor. Long *de novo* MD simulations for 10 ns performed allowed us to evaluate the dynamic behaviour of the system studied and corroborate the docking results, as well as identify key residues in the enzyme-inhibitor interactions. The present MD simulations support the hypothesis that WA is a prospective ligand that has potential to target/inhibit DNA polymerase of the Herpes simplex virus. Results of these studies will also guide the design of selective inhibitors of DNA POL with high specificity and potent activity in order to strengthen the therapeutic arsenal available today against the dangerous biological warfare agent represented by Herpes Simplex Virus*.*

## Authors' contributions

AG, VSB and DS designed the methods and experimental setup. AG carried out the implementation of the various methods. AS and VA assisted AG in this process. AG and DS wrote the manuscript. All authors have read and approved the final manuscript.

## Competing interests

The authors declare that they have no competing interests

## References

[B1] Prevention and control of Herpes virus diseases Clinical and laboratory diagnosis and chemotherapy 1985 63 Bulletin of the WHO 182 185

[B2] Whitley RJ Gnann JWJ Roizmann B, Whitley RJ, Lopez C The Human Herpesviruses The Human Herpesviruses 1993 69 105

[B3] Wild K Bohner T Folkers G Schulz GE The structures of thymidine kinase from Herpes simplex virus type 1 in complex with substrates and a substrate analogue Protein Science 1997 6 2097 2106 9336833 10.1002/pro.5560061005 PMC2143568

[B4] Corey L Wald A Patel R Sacks SL Tyring SK Warren T Douglas JM Paavonen J Morrow RA Beutner KR Once-daily valacyclovir to reduce the risk of transmission of genital herpes New England Journal of Medicine 2004 350 11 20 10.1056/NEJMoa035144 14702423

[B5] Siakallis G Spandidos DA Sourvinos G Herpesviridae and novel inhibitors Antiviral Therapy 2009 14 1051 1064 20032535 10.3851/IMP1467

[B6] Coen DM Schaffer PA Antiherpesvirus drugs: a promising spectrum of new drugs and drug targets Nat Rev Drug Discov 2003 2 278 288 10.1038/nrd1065 12669027

[B7] Chatis PA Miller CH Schrager LE Crumpacker CS Successful treatment with foscarnet of an acyclovir-resistant mucocutaneous infection with Herpes-simplex virus in a patient with Acquired Immunodeficiency Syndrome New England Journal of Medicine 1989 320 297 300 10.1056/NEJM198902023200507 2536137

[B8] Erice A Chou S Biron KK Stanat SC Balfour HH Jordan MC Progressive disease due to ganciclovir-resistant cytomegalo-virus in immunocompromised patients New England Journal of Medicine 1989 320 289 293 10.1056/NEJM198902023200505 2536135

[B9] Erlich KS Mills J Chatis P Mertz GJ Busch DF Follansbee SE Grant RM Crumpacker CS Acyclovir-Resistant Herpes-Simplex Virus-Infections in Patients with the Acquired Immunodeficiency Syndrome New England Journal of Medicine 1989 320 293 296 10.1056/NEJM198902023200506 2536136

[B10] Field AK Biron KK The end of innocence revisited - resistance of Herpesviruses to antiviral drugs Clinical Microbiology Reviews 1994 7 1 13 8118786 10.1128/cmr.7.1.1 PMC358302

[B11] Gilbert C Bestman-Smith J Boivin G Resistance of herpesviruses to antiviral drugs: clinical impacts and molecular mechanisms Drug Resist Updat 2002 5 88 114 10.1016/S1368-7646(02)00021-3 12135584

[B12] Hirsch MS Schooley RT Resistance to antiviral drugs: the end of innocence N Engl J Med 1989 320 313 314 10.1056/NEJM198902023200510 2536138

[B13] Kost RG Hill EL Tigges M Straus SE Brief report: recurrent acyclovir-resistant genital herpes in an immunocompetent patient N Engl J Med 1993 329 1777 1782 10.1056/NEJM199312093292405 8232486

[B14] Greco A Diaz JJ Thouvenot D Morfin F Novel targets for the development of anti-herpes compounds Infect Disord Drug Targets 2007 7 11 18 10.2174/187152607780090766 17346207

[B15] Liu S Knafels JD Chang JS Waszak GA Baldwin ET Deibel MR Jr. Thomsen DR Homa FL Wells PA Tory MC Crystal structure of the herpes simplex virus 1 DNA polymerase Journal of Biological Chemistry 2006 281 18193 18200 10.1074/jbc.M602414200 16638752

[B16] Kambizi L Goosen BM Taylor MB Afolayan AJ Anti-viral effects of aqueous extracts of Aloe ferox and Withania somnifera on herpes simplex virus type 1 in cell culture South African Journal of Science 2007 103 359 360

[B17] Verma H Patil PR Kolhapure RM Gopalkrishna V Antiviral activity of the Indian medicinal plant extract, Swertia chirata against herpes simplex viruses: A study by in-vitro and molecular approach Indian Journal of Medical Microbiology 2008 26 322 326 10.4103/0255-0857.43561 18974483

[B18] Widodo N Takagi Y Shrestha BG Ishii T Kaul SC Wadhwa R Selective killing of cancer cells by leaf extract of Ashwagandha: Components, activity and pathway analyses Cancer Letters 2008 262 37 47 10.1016/j.canlet.2007.11.037 18191020

[B19] Mishra L Singh B Dagenias S Scientific basis for the therapeutic use of *Withania somnifera* (ashwagandha): a review Alternative Medicine Review 2000 5 334 336 10956379

[B20] Panda S Kar A Changes in thyroid hormone concentrations after administration of Ashwagandha root extract to adult male mice Journal of Pharmacy and Pharmacology 1998 50 1065 1068 10.1111/j.2042-7158.1998.tb06923.x 9811169

[B21] Budhiraja RD Sudhir S Review of Biological-Activity of Withanolides Journal of Scientific & Industrial Research 1987 46 488 49122025968

[B22] Kulkarni S George B Mathur R Protective effect of *Withania somnifera* root extract on electrographic activity in a lithium pilocarpine model of status epilepticus Phytotherapy Research 1998 12 451 453 10.1002/(SICI)1099-1573(199809)12:6<451::AID-PTR328>3.0.CO;2-C

[B23] Bhattacharya A Ghosal S Bhattacharya SK Anti-oxidant effect of *Withania somnifera* glycowithanolides in chronic footshock stress-induced perturbations of oxidative free radical scavenging enzymes and lipid peroxidation in rat frontal cortex and striatum Journal of Ethnopharmacology 2001 74 1 6 10.1016/S0378-8741(00)00309-3 11137343

[B24] Bhattacharya SK Muruganandam AV Adaptogenic activity of *Withania somnifera*: an experimental study using a rat model of chronic stress Pharmacology Biochemistry and Behavior 2003 75 547 555 10.1016/S0091-3057(03)00110-2 12895672

[B25] Chaudhary G Sharma U Jagannathan NR Gupta YK Evaluation of *Withania somnifera* in a middle cerebral artery occlusion model of stroke in rats Clinical and Experimental Pharmacology and Physiology 2003 30 399 404 10.1046/j.1440-1681.2003.03849.x 12859433

[B26] Mirjalili MH Moyano E Bonfill M Cusido RM Palazon J Steroidal lactones from Withania somnifera, an ancient plant for novel medicine Molecules 2009 14 2373 2393 10.3390/molecules14072373 19633611 PMC6255378

[B27] Berman HM Westbrook J Feng Z Gilliland G Bhat TN Weissig H Shindyalov IN Bourne PE The protein data bank Nucleic Acids Research 2000 28 235 242 10.1093/nar/28.1.235 10592235 PMC102472

[B28] Grover A Shandilya A Agrawal V Pratik P Bhasme D Bisaria VS Sundar D Hsp90/Cdc37 Chaperone/co-chaperone complex, a novel junction anticancer target elucidated by the mode of action of herbal drug Withaferin A BMC Bioinformatics 2011 12 Suppl 1 S30 10.1186/1471-2105-12-S1-S30 21342561 PMC3044286

[B29] NCBI-PubChem Compound database http://pubchem.ncbi.nlm.nih.gov/

[B30] Case DA DT Cheatham TE Simmerling CL Wang J Duke RE Luo R Walker RC Zhang W Merz KM Roberts B Wang B Hayik S Roitberg A Seabra G Kolossváry I Wong IF Paesani F Vanicek J Wu X Brozell SR Steinbrecher T Gohlke H Cai Q Ye X Wang J Hsieh MJ Cui G Roe DR Mathews DH Seetin MG Sagui C Babin V Luchko T Gusarov S Kovalenko A Kollman PA AMBER 11 2010 San Francisco: University of California

[B31] Morris GM Goodsell DS Halliday RS Huey R Hart WE Belew RK Olson AJ Automated docking using a Lamarckian genetic algorithm and an empirical binding free energy function Journal of Computational Chemistry 1998 19 1639 1662 10.1002/(SICI)1096-987X(19981115)19:14<1639::AID-JCC10>3.0.CO;2-B

[B32] DeLano W The PyMOL Molecular Graphics System 2002 2002 San Carlos, CA: DeLano Scientific

[B33] Jain T Jayaram B An all atom energy based computational protocol for predicting binding affinities of protein-ligand complexes Febs Letters 2005 579 6659 6666 10.1016/j.febslet.2005.10.031 16307743

[B34] Wallace AC Laskowski RA Thornton JM LIGPLOT: a program to generate schematic diagrams of protein-ligand interactions Protein Eng 1995 8 127 134 10.1093/protein/8.2.127 7630882

[B35] Brideau RJ Knechtel ML Huang A Vaillancourt VA Vera EE Oien NL Hopkins TA Wieber JL Wilkinson KF Rush BD Broad-spectrum antiviral activity of PNU-183792, a 4-oxo-dihydroquinoline, against human and animal herpesviruses Antiviral Res 2002 54 19 28 10.1016/S0166-3542(01)00208-X 11888654

[B36] Oien NL Brideau RJ Hopkins TA Wieber JL Knechtel ML Shelly JA Anstadt RA Wells PA Poorman RA Huang A Broad-spectrum antiherpes activities of 4-hydroxyquinoline carboxamides, a novel class of herpesvirus polymerase inhibitors Antimicrob Agents Chemother 2002 46 724 730 10.1128/AAC.46.3.724-730.2002 11850254 PMC127502

[B37] Vajpayee M Malhotra N Antiviral drugs against herpes infection Indian Journal of Pharmacology 2000 32 330 338

